# Supplementary material for: The association between long-term opioid therapy and composite infection-related dental outcomes
Source: PLoS One. 2026 Feb 2;21(2):e0341361. doi: 10.1371/journal.pone.0341361 (PMC12863492; doi:10.1371/journal.pone.0341361)
Supplement: S1 Table — (DOCX) [file pone.0341361.s001.docx]

**Supplemental Table 1**. Current dental terminology (CDT) and international classification of diseases (ICD) codes for the composite of infection-related dental outcomes (CIDO), a composite of dental caries, oral infections, and loss of teeth.

| **CDT codes:** |
| --- |
| D2140-D2999: Dental restoration codes |
| D3110-D3999: Root canal therapy codes |
| D4210-D4999: Periodontal therapy codes |
| D5110-5999: Implant, prosthesis |
| D6010-6199: Implant procedure |
| D7111-D7999: Oral surgery codes |
| **ICD codes:** |
| K11.7: Disturbances of salivary secretion |
| K02.63: DENTAL CARIES ON SMOOTH SURFACE PENETRATING INTO PULP |
| K02.7: DENTAL ROOT CARIES |
| K02.9: DENTAL CARIES UNSPECIFIED |
| K04.4 - ACUTE APICAL PERIODONTITIS OF PULPAL ORIGIN |
| K04.6 - PERIAPICAL ABSCESS WITH SINUS |
| K05.20 - AGGRESSIVE PERIODONTITIS, UNSPECIFIED |
| K05.211 - AGGRESSIVE PERIODONTITIS, LOCALIZED, SLIGHT |
| K05.212 - AGGRESSIVE PERIODONTITIS, LOCALIZED, MODERATE |
| K05.213 - AGGRESSIVE PERIODONTITIS, LOCALIZED, SEVERE |
| K05.219 -AGGRESSIVE PERIODONTITIS, LOCALIZED, UNSPECIFIED SEVERITY |
| K05.221 - AGGRESSIVE PERIODONTITIS, GENERALIZED, SLIGHT |
| K05.222 – AGGRESSIVE PERIODONTITIS, GENERALIZED, MODERATE |
| K13: OTHER DISEASES OF LIP AND ORAL MUCOSA |
| 521.02 - DENTAL CARIES EXTENDING INTO DENTINE |
| 521.03 - DENTAL CARIES EXTENDING INTO PULP |
| 521.08 -DENTAL CARIES OF ROOT SURFACE |
| 521.3 - EROSION, UNSPECIFIED |
| 522 - PULPITIS |
| 522.1 - NECROSIS OF THE PULP |
| 522.4 - ACUTE APICAL PERIODONTITIS OF PULPAL ORIGIN |
| 522.5 - PERIAPICAL ABSCESS WITHOUT SINUS |
| 523 - ACUTE GINGIVITIS, PLAQUE INDUCED |
| 523.1 - CHRONIC GINGIVITIS, PLAQUE INDUCED |
| 523.33 - ACUTE PERIODONTITIS |
| 523.4 - CHRONIC PERIODONTITIS, UNSPECIFIED |
| 523.41 - CHRONIC PERIODONTITIS, LOCALIZED |
| 523.42 - CHRONIC PERIODONTITIS, GENERALIZED |
| 524.6 - TEMPOROMANDIBULAR JOINT DISORDER, UNSPECIFIED |
| 525.12 - LOSS OF TEETH DUE TO PERIODONTAL DISEASE |
| 525.13 - LOSS OF TEETH DUE TO CARIES |
| 525.19 - OTHER LOSS OF TEETH |
| 525.3 - RETAINED DENTAL ROOT |
| 526.2 - OTHER CYSTS OF JAWS |
| 526.4 - INFLAMMATORY CONDITIONS OF JAW |
| 526.5 - ALVEOLITIS OF JAW |
| 527.2 - SIALOADENITIS |
| 527.5 - SIALOLITHIASIS |
| 528.09 - OTHER STOMATITIS AND MUCOSITIS (ULCERATIVE) |
| 528.2 - ORAL APHTHAE |
| 528.3 - CELLULITIS AND ABSCESS OF ORAL SOFT TISSUES |
| 528.4 - CYSTS OF ORAL SOFT TISSUES |
