# Supplementary material for: The association between long-term opioid therapy and composite infection-related dental outcomes
Source: PLoS One. 2026 Feb 2;21(2):e0341361. doi: 10.1371/journal.pone.0341361 (PMC12863492; doi:10.1371/journal.pone.0341361)
Supplement: Attachment — Submitted filename: PONE-D-25-46805.pdf [file pone.0341361.s002.pdf]

PLOS ONE

The association of long-term opioid therapy and dental disease

--Manuscript Draft--

**Manuscript Number:**	PONE-D-25-46805
**Article Type:**	Research Article
**Full Title:**	The association of long-term opioid therapy and dental disease
**Short Title:**	Opioid therapy and dental disease

**Corresponding Author:**	Anne C Black, PhD<br>Yale School of Medicine: Yale University School of Medicine<br>West Haven, CT UNITED STATES OF AMERICA

**Keywords:**	opioids, opioid use disorder, dental disease, Veterans, chronic pain

Abstract:

Background

The Food and Drug Administration's warning that transmucosal buprenorphine, a partial opioid agonist used to treat opioid use disorder and chronic pain, may cause dental disease opens questions about potential class-wide adverse effects involving more widely prescribed opioid analgesics.

Methods

This was a retrospective matched national cohort study. Cox regression models estimated the association between LTOT and a composite infection-related dental outcome (CIDO). Sensitivity analyses excluded patients with cancer, restricted to patients with comprehensive dental coverage, and to patients with ≥180 days of follow-up time, respectively.

Use full form

The national cohort included patients in care at the Department of Veterans Affairs (VA) between October 2010-September 2019. Patients prescribed LTOT were matched 1:2 to patients not prescribed LTOT on age, sex, service region, and VA dental coverage.

Results

The matched cohort of 2,173,435 patients included 787,825 (36%) receiving LTOT; 612,101 (28%) experienced CIDO. In both simple and multivariable regression models, LTOT exposure was associated with greater CIDO risk; HR (95% CI) =1.24 (1.23, 1.25); aHR (95% CI) =1.08 (1.07, 1.09), respectively; p <0.001. Sensitivity analyses showed similar results except among patients with full dental coverage for whom CIDO rates were substantially higher and LTOT was not statistically significantly associated with risk.

Conclusions

In a large national cohort of patients in VA care, LTOT was associated with CIDO. High CIDO rates among patients with full VA dental coverage reflect their unique vulnerability to dental infection associated with service-related conditions. Results may inform patient and provider decision-making around use of LTOT.

Order of Authors:

Anne C Black, PhD

Kirsha S. Gordon, PhD, MS

James D. Dziura, PhD

Declan T. Barry, PhD

Stephen Crystal, PhD

E. Jennifer Edelman, MD, MHS

Gretchen Gibson, DDS, MPH

Powered by Editorial Manager® and ProduXion Manager® from Aries Systems Corporation

	Michelle Hamilton, DMD, PhD
	M. Marianne Jurassic, DMD, MPH
	Yu Li, MD, PhD
	Brandon DL Marshall, PhD
	Melissa Skanderson, MSW
	Katie Suda, PharmD, MS
	William C. Becker, MD
**Additional Information:**	
**Question**	**Response**
**Financial Disclosure**<br><br>Enter a financial disclosure statement that describes the sources of funding for the work included in this submission. Review the submission guidelines for detailed requirements. View published research articles from *PLOS ONE* for specific examples.<br><br>This statement is required for submission and **will appear in the published article** if the submission is accepted. Please make sure it is accurate.<br><br>**Funded studies**<br>Enter a statement with the following details:<br>• Initials of the authors who received each award<br>• Grant numbers awarded to each author<br>• The full name of each funder<br>• URL of each funder website<br>• Did the sponsors or funders play any role in the study design, data collection and analysis, decision to publish, or preparation of the manuscript?<br><br>Did you receive funding for this work?	Yes
Please add funding details.<br>as follow-up to "**Financial Disclosure**<br><br>Enter a financial disclosure statement that describes the sources of funding for the work included in this submission. Review the submission guidelines for detailed requirements. View published research articles from *PLOS ONE* for specific	The study was funded in part by the following:<br>National Institute on Drug Abuse, 1RM1DA055310-01 to WCB. Website: https://nida.nih.gov/ Link to the specific grant: https://reporter.nih.gov/search/ykRq_72sLUux956JebQmtQ/project-details/10652027<br>The funders played no role in study design, data collection or analysis or publishing decision.<br><br>National Institute on Alcohol Abuse and Alcoholism, P01 AA029545-4 to ACJ (not an author). Website: https://niaaa.org/ Link to the specific grant:

examples.<br><br>This statement is required for submission and **will appear in the published article** if the submission is accepted. Please make sure it is accurate.<br><br>**Funded studies**<br>Enter a statement with the following details:<br>• Initials of the authors who received each award<br>• Grant numbers awarded to each author<br>• The full name of each funder<br>• URL of each funder website<br>• Did the sponsors or funders play any role in the study design, data collection and analysis, decision to publish, or preparation of the manuscript?<br><br>Did you receive funding for this work?"	https://reporter.nih.gov/search/nEMK3R2Zl0KdHeZJ5l4evA/project-details/10906187 The funders played no role in study design, data collection or analysis or publishing decision.<br><br>National Institute on Alcohol Abuse and Alcoholism, U24 AA020794 to ACJ (not an author). Website: https://niaaa.org/ Link to the specific grant: https://reporter.nih.gov/search/5j6Au2pzsU2wWc_DYZozFA/project-details/10003103 The funders played no role in study design, data collection or analysis or publishing decision.<br><br>National Institute on Alcohol Abuse and Alcoholism, U10 AA013566 to ACJ (not an author). Website: https://niaaa.org/ Link to the specific grant: https://reporter.nih.gov/search/rJptGFAHH0yEMvvlAHKwkw/project-details/7682335 The funders played no role in study design, data collection or analysis or publishing decision.
Please select the country of your main research funder (please select carefully as in some cases this is used in fee calculation).<br>as follow-up to "**Financial Disclosure**<br><br>Enter a financial disclosure statement that describes the sources of funding for the work included in this submission. Review the submission guidelines for detailed requirements. View published research articles from *PLOS ONE* for specific examples.<br><br>This statement is required for submission and **will appear in the published article** if the submission is accepted. Please make sure it is accurate.	UNITED STATES - US

Powered by Editorial Manager® and ProduXion Manager® from Aries Systems Corporation

**Funded studies**<br>Enter a statement with the following details:<br>• Initials of the authors who received each award<br>• Grant numbers awarded to each author<br>• The full name of each funder<br>• URL of each funder website<br>• Did the sponsors or funders play any role in the study design, data collection and analysis, decision to publish, or preparation of the manuscript?<br><br>Did you receive funding for this work?"	
**Competing Interests**<br><br>Use the instructions below to enter a competing interest statement for this submission. On behalf of all authors, disclose any competing interests that could be perceived to bias this work—acknowledging all financial support and any other relevant financial or non-financial competing interests.<br><br>This statement is required for submission and **will appear in the published article** if the submission is accepted. Please make sure it is accurate and that any funding sources listed in your Funding Information later in the submission form are also declared in your Financial Disclosure statement.<br><br>View published research articles from *PLOS ONE* for specific examples.	The authors have declared that no competing interests exist.

Powered by Editorial Manager® and ProduXion Manager® from Aries Systems Corporation

**NO authors have competing interests**<br><br>Enter: *The authors have declared that no competing interests exist.*<br><br>**Authors with competing interests**<br><br>Enter competing interest details beginning with this statement:<br><br>*I have read the journal's policy and the authors of this manuscript have the following competing interests: [insert competing interests here]*<br><br>* typeset	
**Ethics Statement**<br><br>Enter an ethics statement for this submission. This statement is required if the study involved:<br><br>• Human participants<br>• Human specimens or tissue<br>• Vertebrate animals or cephalopods<br>• Vertebrate embryos or tissues<br>• Field research<br><br>Write "N/A" if the submission does not require an ethics statement.<br><br>General guidance is provided below. Consult the submission guidelines for detailed instructions. **Make sure that all information entered here is included in the Methods section of the manuscript.**	This study was conducted under waiver of informed consent with approval by the VA Connecticut Healthcare System Research and Development Committee and Yale University Human Investigation Committee. Data were accessed for this study between January 24, 2024-March 28, 2025. Authors with IRB-approved access to the data (ACB, KSG, MS, WCB) had access to information that could identify individual participants during and after the data access period.

Powered by Editorial Manager® and ProduXion Manager® from Aries Systems Corporation

**Format for specific study types**<br><br>**Human Subject Research (involving human participants and/or tissue)**<br>• Give the name of the institutional review board or ethics committee that approved the study<br>• Include the approval number and/or a statement indicating approval of this research<br>• Indicate the form of consent obtained (written/oral) or the reason that consent was not obtained (e.g. the data were analyzed anonymously)<br><br>**Animal Research (involving vertebrate animals, embryos or tissues)**<br>• Provide the name of the Institutional Animal Care and Use Committee (IACUC) or other relevant ethics board that reviewed the study protocol, and indicate whether they approved this research or granted a formal waiver of ethical approval<br>• Include an approval number if one was obtained<br>• If the study involved *non-human primates*, add *additional details* about animal welfare and steps taken to ameliorate suffering<br>• If anesthesia, euthanasia, or any kind of animal sacrifice is part of the study, include briefly which substances and/or methods were applied<br><br>**Field Research**<br><br>Include the following details if this study involves the collection of plant, animal, or other materials from a natural setting:<br>• Field permit number<br>• Name of the institution or relevant body that granted permission	
**Data Availability**<br><br>Authors are required to make all data underlying the findings described fully available, without restriction, and from the time of publication. PLOS allows rare exceptions to address legal and ethical concerns. See the PLOS Data Policy and FAQ for detailed information.	No - some restrictions will apply

A Data Availability Statement describing where the data can be found is required at submission. Your answers to this question constitute the Data Availability Statement and **will be published in the article**, if accepted.<br><br>**Important:** Stating 'data available on request from the author' is not sufficient. If your data are only available upon request, select 'No' for the first question and explain your exceptional situation in the text box.<br><br>Do the authors confirm that all data underlying the findings described in their manuscript are fully available without restriction?	
**Describe where the data may be found in full sentences. If you are copying our sample text, replace any instances of XXX with the appropriate details.**<br><br>• If the data are **held or will be held in a public repository**, include URLs, accession numbers or DOIs. If this information will only be available after acceptance, indicate this by ticking the box below. For example: *All XXX files are available from the XXX database (accession number(s) XXX, XXX.).*<br>• If the data are all contained **within the manuscript and/or Supporting Information files**, enter the following: *All relevant data are within the manuscript and its Supporting Information files.*<br>• If neither of these applies but you are able to provide **details of access elsewhere**, with or without limitations, please do so. For example:<br><br>*Data cannot be shared publicly because of [XXX]. Data are available from the XXX Institutional Data Access / Ethics Committee (contact via XXX) for researchers who meet the criteria for access to confidential data.*<br><br>*The data underlying the results presented in the study are available from (include the name of the third party*	Data cannot be shared publicly because of security restrictions within the Department of Veterans Affairs. Researchers may request access to a deidentified data set from the corresponding author. Researchers who meet the criteria for access to confidential data will be asked to sign a data use agreement. Data will be prepared for sharing in accordance with VA's data security guidelines.

and contact information or URL).<br>• This text is appropriate if the data are owned by a third party and authors do not have permission to share the data.<br><br>* typeset	
Additional data availability information:	

Powered by Editorial Manager® and ProduXion Manager® from Aries Systems Corporation

Manuscript

Click here to access/download;Manuscript;Article PLOS One 9.23.25.docx

Title: The association of long-term opioid therapy and dental disease

Short title: Opioid therapy and dental disease

Anne C. Black PhD[*,¶1,2]; Kirsha S. Gordon PhD, MS[¶,1,2]; James D. Dziura PhD[3,4,5]; Declan T. Barry PhD[6,7]; Stephen Crystal PhD[8,9]; E. Jennifer Edelman MD, MHS[2,5]; Gretchen Gibson DDS, MPH[10,11]; Michelle Hamilton DMD, PhD[12]; M. Marianne Jurassic DMD, MPH[13,14]; Yu Li MD, PhD[15]; Brandon DL Marshall PhD[15]; Melissa Skanderson MSW[1]; Katie Suda PharmD, MS[16,17]; William C. Becker MD[1,2]

1. VA Connecticut Healthcare System, West Haven, CT, USA
2. Yale School of Medicine, Department of Internal Medicine; New Haven, CT USA
3. Yale School of Medicine, Department of Emergency Medicine; New Haven, CT USA
4. Yale Center for Analytical Sciences; New Haven, CT USA
5. Yale School of Public Health; New Haven, CT USA
6. Yale School of Medicine; Department of Psychiatry; New Haven, CT USA
7. APT Foundation; New Haven, CT USA
8. Rutgers University, School of Social Work, New Brunswick, NJ USA
9. Rutgers University, Institute for Health, Health Care Policy, and Aging Research, New Brunswick, NJ USA
10. Kansas City University, College of Dental Medicine, Kansas City, MO USA
11. VA Healthcare System of the Ozarks, Fort Smith, AR USA
12. VA Office of Dentistry, Washington DC USA
13. Boston University Goldman School of Dental Medicine, Boston, MA USA
14. VA Bedford Healthcare System, Bedford, MA USA

15. Brown University School of Public Health, Providence, RI USA

16. University of Pittsburgh, School of Medicine, Pittsburgh, PA USA

17. VA Pittsburgh Healthcare System, Pittsburgh, PA USA

*Corresponding author
Email: anne.black@yale.edu (ACB)

¶ These authors contributed equally to this work

Author Contributions: Drs. Gordon and Ms. Skanderson had full access to all of the data in the study and take responsibility for the integrity of the data and the accuracy of the data analysis. **Study concept and design:** Drs. Black, Gordon, Dziura, Gibson, Hamilton, Jurassic, Suda, Becker. **Acquisition, analysis, and interpretation of data:** Drs. Black, Gordon, Becker, and Ms. Skanderson. **Drafting of the manuscript:** Drs. Black, Gordon, Becker. **Critical revision of the manuscript for important intellectual content:** Drs. Barry, Crystal, Dziura, Edelman, Gibson, Hamilton, Jurassic, Li, Marshall, Suda, and Ms. Skanderson. **Statistical analysis:** Drs. Gordon and Dziura. **Obtained funding:** Drs. Gordon, Edelman, and Becker. **Administrative, technical, or material support:** None. **Study supervision:** Dr. Becker.

Abstract

Background: The Food and Drug Administration's warning that transmucosal buprenorphine, a partial opioid agonist used to treat opioid use disorder and chronic pain, may cause dental disease opens questions about potential class-wide adverse effects involving more widely prescribed opioid analgesics.

divide into two sentence for better understanding

Methods: This was a retrospective matched national cohort study. Cox regression models estimated the association between LTOT and a composite infection-related dental outcome (CIDO). Sensitivity analyses excluded patients with cancer, restricted to patients with comprehensive dental coverage, and to patients with ≥180 days of follow-up time, respectively. The national cohort included patients in care at the Department of Veterans Affairs (VA) between October 2010-September 2019. Patients prescribed LTOT were matched 1:2 to patients not prescribed LTOT on age, sex, service region, and VA dental coverage.

Results: The matched cohort of 2,173,435 patients included 787,825 (36%) receiving LTOT; 612,101 (28%) experienced CIDO. In both simple and multivariable regression models, LTOT exposure was associated with greater CIDO risk; HR (95% CI) =1.24 (1.23, 1.25); aHR (95% CI) =1.08 (1.07, 1.09), respectively; $p < 0.001$. Sensitivity analyses showed similar results except among patients with full dental coverage for whom CIDO rates were substantially higher and LTOT was not statistically significantly associated with risk.

Conclusions: In a large national cohort of patients in VA care, LTOT was associated with CIDO. High CIDO rates among patients with full VA dental coverage reflect their unique vulnerability to dental infection associated with service-related conditions. Results may inform patient and provider decision-making around use of LTOT.

Introduction

Clinical guidelines for chronic pain management de-emphasize use of long-term full-opioid agonist therapy (LTOT), such as oxycodone and hydrocodone, given risks for opioid-related harms including opioid use disorder (OUD) and equivocal evidence for long-term effectiveness.[1, 2] For individuals prescribed LTOT with evidence of OUD, guidelines recommend transition to buprenorphine,[3] a partial opioid agonist with analgesic effects and well-established effectiveness for OUD management. Transition to buprenorphine is preferred to drastic reduction or discontinuation of LTOT given the associated high risk for overdose and death.[4] However, in 2022, the Food and Drug Administration[5] published a warning about dental problems associated with transmucosal buprenorphine (e.g., Suboxone, a sublingual buprenorphine/naloxone film), the commonly prescribed buprenorphine formulation, citing risks of tooth decay, cavities, oral infections, and tooth loss. Concern over these risks may present a barrier to buprenorphine initiation in patients prescribed LTOT for whom such treatment is indicated. However, full opioid agonists themselves may pose oral health risks due to immunosuppression and well-documented effects on saliva flow causing xerostomia; both create opportunity for oral disease development.[6, 7] Nevertheless, to our knowledge, no published studies have specifically addressed the dental risks of LTOT. The goal of the current study was to assess the effect of LTOT exposure on dental disease development in a retrospective cohort of patients receiving care within the Department of Veterans Affairs (VA).

Methods

This study was conducted under waiver of informed consent with approval by the VA Connecticut Healthcare System Research and Development Committee and Yale University

Human Investigation Committee. Data were accessed for this study between January 24, 2024-March 28, 2025. Authors with IRB-approved access to the data (ACB, KSG, MS, WCB) had access to information that could identify individual participants during and after the data access period.

Using a national cohort curated by the Veterans Aging Cohort Study (VACS), we created a matched cohort of patients who received VA primary care between October 1, 2010-September 30, 2019 (i.e. fiscal years [FY] 2011-2019) and had not experienced the primary outcome in the year prior to cohort entry. The dataset included all administrative, pharmacy, and healthcare data in VA electronic health records (EHR) for the study period. Patients with incident LTOT prescription (i.e., ≥ 90 consecutive days, allowing for 30-day refill gaps, of hydrocodone, oxycodone, morphine, fentanyl, hydromorphone, dihydrocodeine, meperidine, pentazocine, propoxyphene, levorphanol, tramadol, or tapentadol [excluding methadone], with no exposure to these medications in the past year[8]) were matched 1:2 to patients not prescribed LTOT on age (in 5-year increments), sex, service region, and VA dental coverage. The index date for LTOT-exposed patients was LTOT initiation, and for those not LTOT-exposed, was the first VA visit date within the same FY as matched LTOT-exposed patients. The baseline period was 12 months prior to index date, during which patient characteristics were ascertained.

The primary outcome was the composite infection-related dental outcome (CIDO), a dichotomous indicator of any new-onset dental caries, oral infections, or tooth loss according to International Classification of Diseases (ICD) and Current Procedural Terminology codes (Supplemental Table 1).

We calculated descriptive statistics for the cohort overall and by LTOT exposure and outcome. Group differences were assessed by chi-square, t-tests, or Wilcoxon signed-rank tests.

We used Cox proportional hazards modeling, accounting for matching, to estimate the unconditional effect of LTOT exposure on days to first recorded CIDO, death, or end of study. We then estimated a multivariable Cox model, adjusting for age at index date, race/ethnicity (Black, Hispanic, White, or other), rurality of home address (rural/highly rural vs. not), smoking status (never, unknown, current, past), obesity (body mass index ≥30[9]), pain level (none, non-chronic, or chronic, based on pain-related diagnosis[8]) serious mental health condition (major depressive disorder, post-traumatic stress disorder, bipolar disorder, schizophrenia), substance use (drug use disorder, alcohol use disorder), medical conditions (cancer, chronic obstructive pulmonary disease, stroke, diabetes, human immunodeficiency virus [HIV], hepatitis C [HCV], cardiovascular disease, liver disease), and overall illness severity (measured by the VACS index[10]). Patients with incomplete data and those who died during the baseline period were excluded. Pain, mental health, and medical conditions were determined by ICD codes.

We conducted several sensitivity analyses: (1) excluding patients with cancer, (2) restricting to patients with comprehensive VA dental coverage provided for service-connected dental disability or other disabling medical condition, and (3) restricting to patients with ≥180 days of follow-up time.

Results

The cohort, comprising 2,173,435 patients, was predominantly male (92%) and White (67%), consistent with the VA patient population, and 38% lived in a rural setting. The majority smoked or had smoked (69%), approximately half had obesity (51%) and chronic pain (46%; Table 1). Consistent with the matching schema, approximately one-third (36%) were prescribed LTOT. A total of 28% experienced CIDO.

Table 1. Patients in care FY 2010-2019 characteristics, n=2,173,435

	**Overall**	**Exposure**			**Outcome**		
	**Mean (SD) or N (%)**	**LTOT, n=787825**	**No LTOT, n=1385610**	**p value**	**CIDO, n=612101**	**no CIDO, n=1561334**	**p value**
**Age**, mean (SD)	58 (13)	57 (13)	58 (13)	<0.001	56 (12)	58 (13)	<0.001
**Sex on record**				<0.001			<0.001
Female	162172 (7.46)	60352 (7.66)	101820 (7.35)		51145 (8.36)	111027 (7.11)	
Male	2011263 (92.54)	727473 (92.34)	1283790 (92.65)		560956 (91.64)	1450307 (92.89)	
**Race/Ethnicity**				<0.001			<0.001
Black	413119 (19.01)	132543 (16.82)	280576 (20.25)		149641 (24.45)	263478 (16.88)	
Hispanic	140737 (6.48)	45824 (5.82)	94913 (6.85)		45813 (7.48)	94924 (6.08)	
Other	158499 (7.29)	50880 (6.46)	107619 (7.77)		40097 (6.55)	118402 (7.58)	
White	1461080 (67.22)	558578 (70.90)	902502 (65.13)		376550 (61.52)	1084530 (69.46)	

**Smoking status**				<0.001			<0.001
Never	662620 (30.49)	191098 (24.26)	471522 (34.03)		178186 (29.11)	484434 (31.03)	
Current	786588 (36.19)	357329 (45.36)	429259 (30.98)		249407 (40.75)	537181 (34.41)	
Past	718448 (33.06)	238132 (30.23)	480316 (34.66)		183800 (30.03)	534648 (34.24)	
unknown/missing	5779 (0.27)	1266 (0.16)	4513 (0.33)		708 (0.12)	5071 (0.32)	
**Urban/Rural**				<0.001			<0.001
Urban	1355513 (62.37)	464232 (58.93)	891281 (64.32)		401671 (65.62)	953842 (61.09)	
Rural	784145 (36.08)	309918 (39.34)	474227 (34.23)		202075 (33.01)	582070 (37.28)	
Highly rural	30133 (1.39)	12294 (1.56)	17839 (1.29)		7474 (1.22)	22659 (1.45)	
unknown/missing	3644 (0.27)	1381 (0.18)	2263 (0.16)		881 (0.14)	2763 (0.18)	
**Pain level**				<0.001			<0.001
none	1094435 (50.36)	196020 (24.88)	898415 (64.84)		253741 (41.45)	840694 (53.84)	
Non chronic	68301 (3.14)	22411 (2.84)	45890 (3.31)		20244 (3.31)	48057 (3.08)	

Chronic	1010699 (46.50)	569394 (72.27)	441305 (31.85)		338116 (55.24)	672583 (43.08)	
**Mental health conditions**							
Bipolar disorder	58486 (2.69)	29168 (3.70)	29318 (2.12)	<0.001	27525 (4.50)	30961 (1.98)	<0.001
Major depression	168053 (7.73)	91634 (11.63)	76419 (5.52)	<0.001	71579 (11.69)	96474 (6.18)	<0.001
PTSD	289356 (13.31)	139683 (17.73)	149673 (10.80)	<0.001	147119 (24.04)	142237 (9.11)	<0.001
Schizophrenia	36505 (1.68)	11442 (1.45)	25063 (1.81)	<0.001	20287 (3.31)	16218 (1.04)	<0.001
SMH (any of the above)	449048 (20.66)	217078 (27.55)	231970 (16.74)	<0.001	211432 (34.54)	237616 (15.22)	<0.001
Stroke	32758 (1.51)	15937 (2.02)	16821 (1.21)	<0.001	11106 (1.81)	21652 (1.39)	<0.001
COPD	149661 (6.89)	86921 (11.03)	62740 (4.53)	<0.001	47862 (7.82)	101799 (6.52)	<0.001
AUD	160406 (7.38)	72381 (9.19)	88025 (6.35)	<0.001	70794 (11.57)	89612 (5.74)	<0.001
Drug use disorder	109163 (5.02)	53906 (6.84)	55257 (3.99)	<0.001	54854 (8.96)	54309 (3.48)	<0.001
OUD	20501 (0.94)	12219 (1.55)	8282 (0.60)	<0.001	10358 (1.69)	10143 (0.65)	<0.001
Obesity (BMI ≥30)	1102421 (50.72)	416598 (52.88)	685823 (49.50)	<0.001	321561 (52.53)	780860 (50.01)	<0.001

Diabetes	476855 (21.94)	207014 (26.28)	269841 (19.47)	<0.001	155648 (25.43)	321207 (20.57)	<0.001
HCV	66863 (3.08)	36332 (4.61)	30531 (2.20)	<0.001	26993 (4.41)	39870 (2.55)	<0.001
HIV	11837 (0.54)	4426 (0.56)	7411 (0.53)	0.01	4955 (0.81)	6882 (0.44)	<0.001
Cancer	122236 (5.62)	60717 (7.71)	61519 (4.44)	<0.001	43051 (7.03)	79185 (5.07)	<0.001
CVD	64301 (2.96)	31805 (4.04)	32496 (2.35)	<0.001	21290 (3.48)	43011 (2.75)	<0.001
Liver disease	63059 (2.90)	34230 (4.34)	28829 (2.08)	<0.001	24051 (3.93)	39008 (2.50)	<0.001
**Dental class**				<0.001			<0.001
unknown/missing	1231043 (56.64)	458060 (58.14)	772983 (55.79)		57401 (9.38)	1173642 (75.17)	
no coverage	619405 (28.50)	218089 (27.68)	401316 (28.96)		382896 (62.55)	236509 (15.15)	
Some coverage	315745 (14.53)	109095 (13.85)	206650 (14.91)		167013 (27.29)	148732 (9.53)	
Comprehensive coverage	7242 (0.33)	2581 (0.33)	4661 (0.34)		4791 (0.78)	2451 (0.16)	
**LTOT**	787825 (36.25)				232944 (38.06)	554881 (35.54)	<0.001
**CIDO**	612101 (28.16)	232944 (29.57)	379157 (27.36)	<0.001			

**Died**	542487 (24.96)	239196 (30.36)	303291 (21.89)	<0.001	155769 (25.45)	386718 (24.77)	<0.001
**Severity of illness**, median(IQR)	32 (26, 40)	34 (27, 42)	32 (26, 39)	<0.001	32 (26, 39)	33 (26, 40)	<0.001
**FU**, median(IQR)	7 (3, 9)	6 (3, 9)	7 (3, 9)	<0.001	3 (1, 5)	8 (5, 10)	<0.001

PTSD=post-traumatic stress disorder, SMH=severe mental health condition, COPD=chronic obstructive pulmonary disease, AUD=alcohol use disorder, OUD=opioid use disorder, HCV=hepatitis C, CVD=cardiovascular disease, LTOT=long-term opioid therapy, CIDO=composite of infection-related dental outcomes, FU=months of follow-up after index

Patients prescribed LTOT were more likely to be White, live in a rural setting, smoke currently, have chronic pain, a serious mental health condition (except schizophrenia), substance use disorder, greater illness severity, and experience CIDO. Although statistically significant, differences in VA dental coverage were negligible due to matching (Table 1).

Patients with CIDO, compared to those without, were more likely to be female, Black, reside in a non-rural setting, smoke currently, have chronic pain, a serious mental health condition, substance use disorder, VA dental coverage, and receive LTOT.

In the simple Cox model, patients prescribed LTOT had greater risk of CIDO compared to those not prescribed LTOT (Table 2); hazard ratio (HR) (95% CI) = 1.24 (1.23, 1.25). Adjusting for covariates, the risk related to LTOT was attenuated but remained statistically significant; adjusted hazard ratio (aHR) (95% CI) = 1.08 (1.07, 1.08).

Covariates associated with greater risk for CIDO included having a pain-related diagnosis, serious mental health condition, substance use disorder, cancer diagnosis, and higher illness severity. In contrast, increased age, White or other race (compared to Black), rural residence, HCV, and HIV were associated with lower CIDO risk (Table 2).

Table 2. Cox proportional models of the association between LTOT and CIDO, n=2,173,435

	Unadjusted		Adjusted	
	**HR (95%)**	**p value**	**aHR (95%)**	**p value**
**LTOT**	1.24 (1.23, 1.25)	<0.001	1.08 (1.07, 1.08)	<0.001
**Age/10**			0.95 (0.92, 0.97)	<0.001
**Race/Ethnicity** (ref Black)				
Hispanic			1.01 (0.99, 1.02)	0.42
Other			0.93 (0.92, 0.95)	<0.001
White			0.98 (0.97, 0.99)	<0.001
**Smoking** (ref never)				
Current			1.03 (1.02, 1.04)	<0.001
Past			1.00 (0.99, 1.01)	0.70
Unknown/missing			1.33 (1.18, 1.50)	<0.001
**Rurality**			0.98 (0.97, 0.99)	<0.001
**Pain level** (ref none)				
Non chronic			1.17 (1.16, 1.18)	<0.001
Chronic			1.11 (1.09, 1.14)	<0.001
**SMH**			1.43 (1.41, 1.44)	<0.001
**Stroke**			1.00 (0.96, 1.04)	0.98
**COPD**			1.05 (1.03, 1.07)	<0.001
**AUD**			1.09 (1.07, 1.10)	<0.001
**Drug use disorder**			1.20 (1.18, 1.22)	<0.001

**OUD**			1.01 (0.98, 1.05)	0.54
**BMI>30** (Obesity)			1.02 (1.01, 1.02)	0.0002
**Diabetes**			1.08 (1.07, 1.09)	<0.001
**HCV**			0.93 (0.91, 0.96)	<0.001
**HIV**			0.95 (0.91, 0.99)	0.02
**Cancer**			1.31 (1.29, 1.34)	<0.001
**CVD**			1.07 (1.03, 1.10)	<0.001
**Liver disease**			1.01 (0.99, 1.04)	0.34
**Severity of illness/10**			1.12 (1.12, 1.13)	<0.001

SMH=severe mental health condition (composite of major depression, PTSD, bipolar disorder, schizophrenia), COPD=chronic obstructive pulmonary disease, AUD=alcohol use disorder, HCV=hepatitis C, CVD=cardiovascular disease, LTOT=long-term opioid therapy, CIDO=composite of infection-related dental outcomes

In individual sensitivity analyses limited to patients without cancer, and to those with ≥180-days of follow-up, respectively, results essentially replicated the multivariable full-cohort model's; aHR (95% CI) = 1.08 (1.07, 1.09) (Table 3). In contrast, among patients with comprehensive VA dental coverage, whose CIDO rates were substantially higher than among the cohort overall, there was no association between CIDO and LTOT; aHR (95% CI) = 0.97 (0.89, 1.05).

Table 3. Sensitivity analyses: Model 1 excluded those with cancer, Model 2 restricted to those with comprehensive dental coverage, Model 3 restricted to those with index date ≥180 days prior to end of study

	**Model 1,** n=2,051,199	**Model 2,** n=7242	**Model 3,** n=2,156,900

	HR (95%)	p value	HR (95%)	p value	HR (95%)	p value
**LTOT**	1.08 (1.07, 1.09)	<0.001	0.97 (0.89, 1.05)	0.41	1.08 (1.07, 1.08)	<0.001
**Age/10**	0.94 (0.92, 0.97)	0.0001	0.83 (0.60, 1.13)	0.24	0.95 (0.92, 0.97)	<0.001
**Race/Ethnicity** (ref Black)						
Hispanic	1.00 (0.99, 1.02)	0.65	0.95 (0.78, 1.15)	0.58	1.01 (0.99, 1.02)	0.45
Other	0.93 (0.91, 0.94)	<0.001	0.88 (0.72, 1.06)	0.17	0.93 (0.92, 0.95)	<0.001
White	0.97 (0.96, 0.98)	<0.001	0.91 (0.81, 1.03)	0.13	0.98 (0.97, 0.99)	<0.001
**Smoking** (ref never)						
Current	1.03 (1.02, 1.05)	<0.001	0.88 (0.79, 0.98)	0.02	1.03 (1.02, 1.04)	<0.001
Past	1.00 (0.99, 1.01)	0.45	0.93 (0.83, 1.04)	0.22	1.00 (0.99, 1.01)	0.69
Unknown/missing	1.31 (1.15, 1.51)	<0.001	1.40 (0.30, 6.46)	0.67	1.33 (1.18, 1.50)	<0.001
**Rurality**	0.98 (0.97, 0.99)	<0.001	0.98 (0.89, 1.08)	0.69	0.98 (0.97, 0.99)	<0.001
**Pain level** (ref none)						
Non chronic	1.17 (1.16, 1.18)	<0.001	1.21 (1.10, 1.33)	0.0002	1.17 (1.16, 1.18)	<0.001
Chronic	1.11 (1.09, 1.14)	<0.001	1.22 (0.93, 1.59)	0.15	1.11 (1.09, 1.14)	<0.001
**SMH**	1.43 (1.42, 1.45)	<0.001	1.22 (1.10, 1.36)	0.0002	1.43 (1.41, 1.44)	<0.001
**Stroke**	0.98 (0.94, 1.03)	0.41	1.82 (0.88, 3.78)	0.11	1.00 (0.96, 1.04)	0.94
**COPD**	1.05 (1.03, 1.07)	<0.001	1.16 (0.93, 1.46)	0.19	1.05 (1.03, 1.07)	<0.001
**AUD**	1.09 (1.07, 1.10)	<0.001	1.11 (0.90, 1.37)	0.32	1.09 (1.07, 1.10)	<0.001
**Drug use disorder**	1.20 (1.18, 1.23)	<0.001	1.03 (0.78, 1.35)	0.84	1.20 (1.18, 1.22)	<0.001
**OUD**	1.02 (0.98, 1.06)	0.36	0.80 (0.47, 1.35)	0.40	1.01 (0.98, 1.05)	0.52
**BMI30** (Obesity)	1.02 (1.01, 1.03)	0.000	0.92 (0.84, 1.01)	0.08	1.02 (1.01, 1.03)	0.00

**Diabetes**	1.09 (1.08, 1.10)	<0.001	0.97 (0.85, 1.11)	0.68	1.08 (1.07, 1.09)	<0.001
**HCV**	0.94 (0.91, 0.97)	<0.001	1.05 (0.74, 1.50)	0.78	0.93 (0.91, 0.96)	<0.001
**HIV**	0.97 (0.92, 1.01)	0.17	1.02 (0.52, 2.01)	0.96	0.95 (0.91, 0.99)	0.02
**Cancer**			1.16 (0.92, 1.45)	0.21	1.31 (1.29, 1.33)	<0.001
**CVD**	1.10 (1.06, 1.14)	<0.001	0.66 (0.40, 1.07)	0.09	1.07 (1.03, 1.10)	<0.001
**Liver disease**	1.02 (0.99, 1.04)	0.31	0.93 (0.65, 1.33)	0.68	1.01 (0.99, 1.04)	0.35
**Severity of illness/10**	1.12 (1.11, 1.13)	<0.001	1.00 (0.93, 1.07)	0.97	1.12 (1.12, 1.13)	<0.001

SMH=severe mental health condition (composite of major depression, PTSD, bipolar disorder, schizophrenia), COPD=chronic obstructive pulmonary disease, AUD=alcohol use disorder, HCV=hepatitis C, CVD=cardiovascular disease, LTOT=long-term opioid therapy

Discussion

To our knowledge, this is the first study to demonstrate the association between LTOT exposure and dental disease. This finding is important in light of recent warnings of buprenorphine risks that may influence decision-making in the context of chronic pain and/or OUD. Sensitivity analyses suggest LTOT may pose minimal additional risk to patients already extremely vulnerable to dental infection, such as patients provided VA comprehensive dental coverage. Evidence of LTOT-associated dental risks may inform guidelines for patients' oral healthcare while taking LTOT and discussions before initiating LTOT.

The current study is limited by our use of available data in patients' EHR. Patients without VA dental coverage may have CIDO recorded in other systems not reviewed in the current study. However, given our matching paradigm, misclassification likely would not differ by LTOT status. Smaller sample may have masked differences in CIDO rate among Veterans with full dental coverage. Despite covariate adjustment, unmeasured confounders may have

driven observed associations. Nevertheless, the demonstrated association between incident LTOT exposure and new-onset dental disease in this rigorous, large-sample analysis exposes the need for additional study. To inform the critical decision to transition from LTOT to buprenorphine to address OUD, research is needed to assess the relative oral health risks of these medications.

Acknowledgement: This work uses data provided by patients and collected by the Department of Veterans Affairs as part of their care and support. The views and opinions expressed in this manuscript are those of the authors and do not necessarily represent those of the Department of Veterans Affairs or the United States government.

References

1. Department of Veterans Affairs. VA/DoD clinical practice guideline for the use of opioids in the management of chronic pain. Updated September 23, 2024. Accessed March 18, 2025. https://www.healthquality.va.gov/guidelines/pain/cot/
2. Dowell D, Ragan KR, Jones CM, Baldwin GT, Chou R. CDC clinical practice guideline for prescribing opioids for pain - United States, 2022. MMWR Recomm Rep. 2022;71(3):1-95.
3. U.S. Department of Health and Human Services. HHS guide for clinicians on the appropriate dosage reduction or discontinuation of long-term opioid analgesics. Updated October 2019. Accessed March 18, 2025. https://www.hhs.gov/system/files/Dosage_Reduction_Discontinuation.pdf
4. Oliva EM, Bowe T, Manhapra A, et al. Associations between stopping prescriptions for opioids, length of opioid treatment, and overdose or suicide deaths in US veterans: observational evaluation. BMJ. 2020;368:m283.

5. Knopf A. FDA warns about dental problems with buprenorphine medicines dissolved in the mouth. Brown Univ Child Adolesc Psychopharmacol Update. 2022;24(3):8.
6. Götrick B, Åkerman S, Ericson D, Torstenson R, Tobin G. Oral pilocarpine for treatment of opioid-induced oral dryness in healthy adults. J Dent Res. 2004;83(5):393-397. doi:10.1002/cpu.30656
7. Ninković J, Roy S. Role of the mu-opioid receptor in opioid modulation of immune function. Amino Acids. 2013;45(1):9-24.
8. Edelman EJ, Gordon KS, Becker WC, et al. Receipt of opioid analgesics by HIV-infected and uninfected patients. J Gen Intern Med. 2013;28(1):82-90.
9. Centers for Disease Control. CDC Disability and Obesity. Updated December 16, 2024. Accessed October 30, 2024. https://www.cdc.gov/disability-and-health/conditions/obesity.html?CDC_AAref_Val=https://www.cdc.gov/ncbddd/disabilityandhealth/obesity.html
10. Justice AC, Tate JP. Strengths and limitations of the Veterans Aging Cohort Study Index as a measure of physiologic frailty. AIDS Res Hum Retroviruses. 2019;35(11-12):1023-10

Supplementary Table

Click here to access/download
Supporting Information
Supplement.docx

VA Initial Study approval

Click here to access/download
Supporting Information
VA IRB initial approval.pdf

Yale Initial Study approval

Click here to access/download
Supporting Information
Birth Cohort Yale approval.pdf

VA latest IRB approval 2024

Click here to access/download
Supporting Information
Status Update Acknowledgement letter 2024-2025.pdf

Yale updated IRB approval

Click here to access/download

Supporting Information

VACS National approval Yale.pdf
